# Evaluating Photonic Quantum Memristors in Noisy Environments

**DOI:** 10.3390/e28050507

**Published:** 2026-05-01

**Authors:** Jiachao Wang, Wentao Mao, Tengze Yang, Qiming Zhang, Wei Li

**Affiliations:** 1 School of Artificial Intelligence Science and Technology, University of Shanghai for Science and Technology, Shanghai 200093, China2335054421@st.usst.edu.cn (W.M.);; 2 Institute of Photonic Chips, University of Shanghai for Science and Technology, Shanghai 200093, China

**Keywords:** neuromorphic computing, photonic quantum memristor, photon loss, NARMA2 task

## Abstract

While photonic quantum memristors (PQMs) offer promising avenues for neuromorphic computing, their performance is inherently affected by hardware noise, particularly photon loss and phase fluctuations. This study systematically investigates the impact of photon loss and phase fluctuations on PQM dynamics by employing the noisy gates approach, which integrates dissipative effects directly into the device evolution. At the device level, we demonstrate that photon loss alters the dynamic trajectory of individual PQMs. It induces evident deformations in the characteristic pinched hysteresis loops, with the degradation of non-Markovian memory effects being particularly pronounced at shorter integration times. To further evaluate system-level implications, we construct a two-PQM network to execute the NARMA2 time-series prediction task. Under noiseless conditions, the network exhibits strong representation capabilities with a normalized mean square error (NMSE) of 0.0448. However, performance degrades markedly under incoherent evolution; the NMSE increases to 0.1552, 0.2567, and 0.3056 for photon loss probabilities of 0.2, 0.4, and 0.5, respectively. Furthermore, at a high photon loss probability of 0.5, extending the integration time fails to compensate for the degradation and instead exacerbates the prediction error. These findings indicate that photon loss impairs both individual device dynamics and network-level processing, emphasizing the critical need for loss-tolerant architectures in deploying PQM networks.

## 1. Introduction

Over the past few decades, machine learning has become a key driving force in both real-world applications and scientific research [1,2]. Meanwhile, the rapid growth of data-intensive and computation-heavy tasks has exposed the limitations of conventional von Neumann architectures. Owing to the physical separation of memory and processing units, such architectures suffer from inherent efficiency and scalability bottlenecks, thereby motivating the search for alternative computing paradigms [3,4]. Inspired by the mechanisms of brain-like information processing, neuromorphic computing [5,6] provides a new pathway toward efficient and parallel computation. As a physical device platform for such architectures, memristors [7,8] have become a major research focus because of their ability to emulate synaptic plasticity [9,10,11]. To further overcome the performance bottlenecks of classical devices and exploit the advantages of quantum computing [12], quantum memristors have drawn attention and been theoretically and experimentally explored in superconducting circuits [13], trapped ions [14], and photonic systems [15], among other physical platforms. Among these, PQM have shown particularly prominent performance. They have not only been experimentally demonstrated [16,17], but have also been further applied to the construction of quantum reservoir computing systems [18] for executing specific neuromorphic computing tasks, exhibiting significant development potential and broad application prospects.

Compared with other platforms, photonic quantum systems possess the inherent advantage of extremely weak interaction between photons and the environment [19,20]. However, their practical physical implementation is still constrained by hardware noise, such as coupling defects, measurement errors, and predominantly photon losses [21]. Among these, photon loss, as the most common dissipation mechanism, can alter the history-dependent input–output relations of PQMs, degrading their signature pinched hysteresis loops and non-Markovian memory properties. Since device-level degradation directly affects the computational performance of the entire information-processing system, systematically investigating the dynamics of PQMs under incoherent evolution is of great importance for evaluating their practical application potential.

To address the above issues, this work investigates the effects of photon loss and phase fluctuations on PQM dynamics by employing the noisy gates approach, which integrates dissipative effects directly into the device evolution. At the device level, a single PQM is studied to characterize its history-dependent dynamics under these imperfections. Specifically, the analysis focuses on the deformations in the characteristic pinched hysteresis loops induced by different photon loss probabilities and phase noise levels, and further examines how these noise-induced effects evolve with the integration time. At the system level, a PQM network composed of two independent PQMs is constructed and employed in the NARMA2 time-series prediction task to evaluate the impact of photon loss on the network’s information-processing capability. Under noiseless conditions, the network achieves a normalized mean square error (NMSE) of 0.0448, whereas under photon loss conditions with p=0.2,0.4, and 0.5, the NMSE increases to 0.1552, 0.2567, and 0.3056, respectively. Moreover, at a high photon loss probability of 0.5, we further examine the effect of increasing the integration time on the prediction performance. These results indicate that photon loss not only perturbs the non-Markovian dynamical behavior of individual devices, but also further weakens the representation and processing capability of the network, ultimately leading to degraded task performance. This work provides a theoretical basis for understanding and optimizing loss-tolerant PQM architectures in noisy environments.

## 2. Results

**Figure 1:**
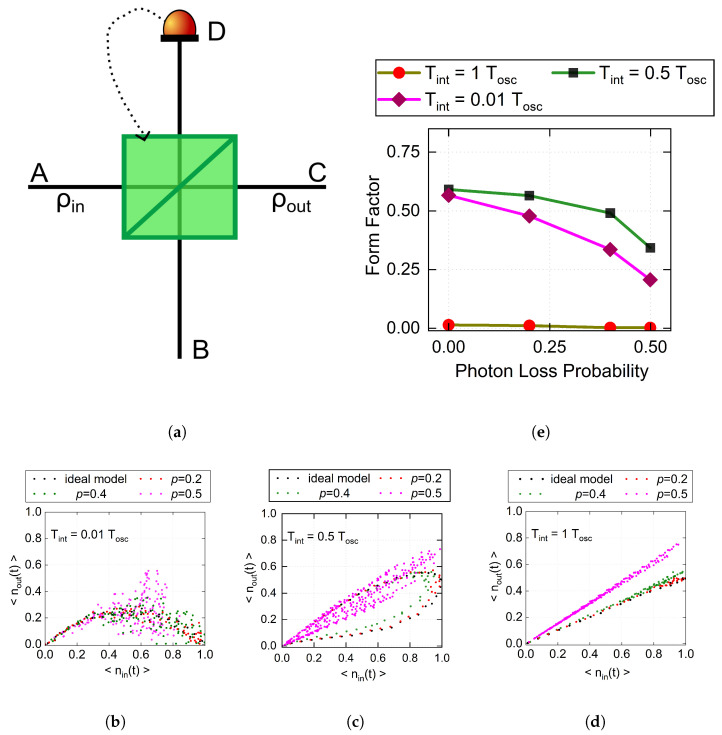
(**a**) Schematic diagram of the photonic memristor structure, where port A, port C, and port D denote the input, output, and feedback ports, respectively. A measurement is performed on port D to update the reflectivity R(t). (**b**–**d**) Nonlinear response curves of the ideal and lossy models for different memory lengths, where each panel shows the responses under different photon loss probabilities (p=0.2, p=0.4, and p=0.5). (**e**) Form factor as a function of photo loss probability for different integration times, where the brown, green, and purple curves denote $T_{\mathrm{int}}=T_{\mathrm{osc}}$, $T_{\mathrm{int}}=0.5\;T_{\mathrm{osc}}$, and $T_{\mathrm{int}}=0.01\;T_{\mathrm{osc}}$, respectively.

A PQM is a quantum system with memory whose response depends on its evolution history while preserving quantum coherence. In this work, the PQM is realized using tunable beam splitters, as shown in Figure 1a. A single-photon state is injected into input port A, while the device reflectivity R(t) is dynamically updated according to the measurement outcome at feedback port D. Let $\langle {\hat{n}}_{\mathrm{in},A}\left(t\right)\rangle$, $\langle {\hat{n}}_{\mathrm{out},C}\left(t\right)\rangle$, and $\langle {\hat{n}}_{D}\left(t\right)\rangle$ denote the mean photon numbers at input port A, output port C, and feedback port D, respectively. The input–output relationship of the device is given by

(1) $$\langle {\hat{n}}_{\mathrm{out},C}\left(t\right)\rangle =\left[1-R\left(t\right)\right]\langle {\hat{n}}_{\mathrm{in},A}\left(t\right)\rangle .$$

The mean photon number at the feedback port is given by $\langle {\hat{n}}_{D}\left(t\right)\rangle =R\left(t\right)\langle {\hat{n}}_{\mathrm{in},A}\left(t\right)\rangle .$ The dynamic update rule for the device reflectivity is given by

(2) $$R\left(t\right)=\frac{1}{T_{\mathrm{int}}}\int_{t-T_{\mathrm{int}}}^{t}\langle {\hat{n}}_{\mathrm{in},A}\left(\tau \right)\rangle d\tau ,$$

where $T_{\mathrm{int}}$ denotes the integration time, which determines the memory extent of the device. Since R(t) depends on the past input within the integration window, the current response is history-dependent, giving rise to memristive behavior.

**Figure 2:**
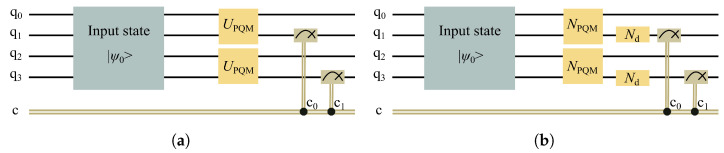
Quantum-circuit implementation of the two-PQM network in Qiskit under (**a**) noiseless conditions and (**b**) photon loss conditions. The input state is initialized at the beginning of the circuit. Under noiseless conditions, the evolution is governed by the unitary gate $U_{\mathrm{PQM}}$, whereas under photon loss conditions, $U_{\mathrm{PQM}}$ is replaced by the noisy gate $N_{\mathrm{PQM}}$ to incorporate photon loss effects, with additional noisy gates introduced in the detection stage. The final measurement completes a single-step evolution. Here, $c_{0}$ and $c_{1}$ store the expected photon numbers at the outputs of the two PQM feedback ports, respectively, for updating the reflectivities of the corresponding quantum memristors.

For numerical simulations, we implement the PQM circuit model on the Qiskit platform under noiseless conditions, as illustrated in Figure 2a. In this ideal model, noise effects are neglected. As a result, the PQM can be regarded as a closed quantum system whose evolution is fully described by a unitary gate. Therefore, for an input state $|\psi_{\mathrm{in}}\rangle$, the output state is given by

(3) $$|\psi_{\mathrm{out}}\rangle \;=\;U_{\mathrm{PQM}}|\psi_{\mathrm{in}}\rangle ,$$

where $U_{\mathrm{PQM}}$ denotes the evolution gate of the PQM.

To characterize the memristive behavior of the PQM, we apply a time dependent input state and study the corresponding system response for different integration times $T_{\mathrm{int}}$. The input state is defined as

(4) $$|\psi \rangle =\cos\frac{\pi t}{T_{\mathrm{osc}}}{|0\rangle }_{A}+\sin\frac{\pi t}{T_{\mathrm{osc}}}{|1\rangle }_{A},$$

where $T_{\mathrm{osc}}$ denotes the oscillation period of the input signal, and its accessible range is constrained by the actual response rate of the device. ${|0\rangle }_{A}$ and ${|1\rangle }_{A}$ denote the vacuum and single-photon Fock states in port A, respectively. The device dynamics are mainly determined by the ratio $T_{\mathrm{int}}/$$T_{\mathrm{osc}}$ [17]. Therefore, different $T_{\mathrm{int}}$ values are chosen to probe the response of the PQM under different memory conditions, as illustrated in Figure 1. These curves show that the system output depends not only on the instantaneous input but also on its history, which is the hallmark of memristive behavior. Specifically, when $T_{\mathrm{int}}=T_{\mathrm{osc}}$, the response curve is linear, indicating a weaker memory effect, whereas for $T_{\mathrm{int}}=0.5\;T_{\mathrm{osc}}$ and $T_{\mathrm{int}}=0.01\;T_{\mathrm{osc}}$, the response becomes nonlinear, corresponding to stronger memory effects.

However, in realistic open quantum systems [22], quantum information processing is inevitably subject to various sources of noise. For photonic platforms, the relevant noise mainly arises from two aspects: first, photon loss caused by absorption or scattering during photon transmission and coupling in optical networks; second, imperfect detection efficiency inherent to single-photon detectors. Collectively, these effects deteriorate the performance of the quantum system.

To describe realistic quantum devices more faithfully, we adopt existing quantum noise modeling methods and map dissipative effects onto quantum noise channels in the evolution process [23]. Actual hardware implementations will encounter additional non-idealities that may introduce discrepancies from our simulated results. For instance, imperfect photon indistinguishability and multi-photon emission from practical single-photon sources can degrade quantum interference visibility. Additionally, unmodeled hardware noise, such as detector dark counts, can inject false-positive signals into the feedback loop, while thermal crosstalk and phase fluctuations in integrated interferometers can cause deviations from the targeted unitary operations. In our analysis, additional hardware imperfections, such as detector dark counts and timing jitter, are considered negligible. State-of-the-art single-photon sources can achieve brightness levels in the megacounts per second (Mcps) regime [24], whereas the dark-count rate of a typical superconducting nanowire single-photon detector is about 100 cps. Therefore, the contribution of dark counts is negligible. On the other hand, to accumulate efficient photon counts, the required integration time is on the order of milliseconds. Since the timing jitter is only about 50 ps, which is many orders of magnitude smaller than this integration timescale, its effect can also be neglected. Among these imperfections, in this work we focus on two representative factors, photon loss and phase fluctuations, and investigate their impacts on the PQM dynamics.

We first focus on photon loss. The gate matrices used to describe photon loss in this work are constructed following Ref. [21]. Specifically, the lossy evolution in the PQM and the detection process are described by $N_{\mathrm{PQM}}^{\left(\mathrm{loss}\right)}$ and $N_{d}^{\left(\mathrm{loss}\right)}$, respectively.

(5) $$\begin{array}{cc} N_{\mathrm{PQM}}^{\left(\mathrm{loss}\right)}= & \left(\begin{array}{cc} \cos\frac{\theta }{2} & i\sin\frac{\theta }{2}\\ i\sin\frac{\theta }{2} & \cos\frac{\theta }{2} \end{array}\right)\;\cdot \;\left(\begin{array}{cc} \cos(\epsilon_{0}I_{C0}) & 0\\ 0 & 1 \end{array}\right)\;\cdot \;\left(\begin{array}{cc} 1 & 0\\ 0 & \cos(\epsilon_{0}I_{S0}) \end{array}\right)\\ \; & \;\cdot \;\left(\begin{array}{cc} 1 & 0\\ 0 & \cos(\epsilon_{0}I_{C1}) \end{array}\right)\;\cdot \;\left(\begin{array}{cc} \cos(\epsilon_{0}I_{S1}) & 0\\ 0 & 1 \end{array}\right),\\ N_{d}^{\left(\mathrm{loss}\right)}= & \left(\begin{array}{cc} 1 & 0\\ 0 & \cos(\epsilon_{1}W) \end{array}\right), \end{array}$$

where θ controls the splitting ratio of the tunable beam splitter. The parameter $\epsilon_{i}$ (i=0,1) is related to the corresponding photon loss probability $p_{i}$ through $\epsilon_{i}=\sqrt{-\log(1-2p_{i})/2}$. Specifically, $p_{0}$ denotes the photon loss probability during the PQM evolution, whereas $p_{1}$ denotes the photon loss probability in the detection stage. In addition, W denotes the Wiener process, and averaging over W yields $E\left[\sin^{2}\left(\epsilon_{1}W\right)\right]=p_{1}$. Similarly, by averaging on the processes $I_{C0}$, $I_{C1}$, $I_{S0}$, and $I_{S1}$, one obtains $E\left[\sin^{2}\left(\epsilon_{0}I_{k}\right)\right]=p_{0}$, with k=C0,C1,S0,S1.

In numerical simulations we introduce a unified photon loss probability by setting $p=p_{0}=p_{1}$. This simplification allows us to systematically examine the impact of photon loss on the system dynamics and task performance. In practical implementations, however, the loss probability at different stages can be calibrated independently according to the actual device loss and detector efficiency.

To assess how photon loss in realistic devices modifies the memristive properties exhibited by the PQM under noiseless conditions, we next construct a noisy PQM circuit model by employing the noisy-gates approach, which integrates dissipative effects directly into the device evolution. Specifically, the original PQM circuit architecture is retained, while each unitary gate is replaced by its corresponding noisy gate, as shown in Figure 2b. In this way, photon loss and detection imperfections are incorporated into the circuit evolution in a consistent manner. Based on this noisy model, we perform noisy-circuit simulations on the Qiskit platform to investigate how photon loss affects the dynamical behavior of an individual PQM. The detailed simulation procedure is summarized in Algorithm 1. Briefly, for each sampled noise instance, the noiseless PQM circuit is mapped to a corresponding noisy circuit by replacing each gate $U^{\left(i\right)}$ with its corresponding noisy gate $N^{\left(i\right)}$. The resulting noisy circuit produces an output state $|\psi_{k}\rangle$, from which the density matrix is constructed as $\rho_{k}\;=\;|\psi_{k}\rangle \langle \psi_{k}|$. The final density matrix is then obtained by averaging over all sampled noise instances.

To investigate how photon loss affects the dynamical behavior of an individual PQM, we perform comparisons at a fixed integration time $T_{\mathrm{int}}$, with the photon loss probability set to p=0.2, 0.4, and 0.5. In the simulations, the case p=0.5 is implemented as p=0.4999 to remain within the applicability range of the present model. It should be noted that, following the formulation in Ref. [21], the current model is restricted to the regime p<0.5. Accordingly, regimes with excessively strong photon loss are beyond the scope of the present study.
**Algorithm 1** Noisy-PQM Circuit Simulation.**Require:** Initial state $|\psi_{0}\rangle$, a noiseless circuit $P=\{U^{\left(1\right)},\ldots ,U^{\left(n_{g}\right)}\}$ composed by $n_{g}$ gates $U^{\left(i\right)}$ and number of samples $N_{\mathrm{sam}}$**Ensure:** Final density matrix $\rho^{\prime }$   1: **for**
k=1 to $N_{\mathrm{sam}}$ **do**   2:    map a noisy circuit $\tilde{P}=\left\{N^{\left(1\right)},\ldots ,N^{\left(n_{g}\right)}\right\}\;\mathrm{on}\;P$   3:    apply the noisy gates $|\psi_{k}\rangle \;=\;N^{\left(n_{g}\right)}...N^{\left(1\right)}|\psi_{0}\rangle$   4:    compute $\rho_{k}\;=\;|\psi_{k}\rangle \langle \psi_{k}|$   5: **end for**   6: $\rho^{\prime }=\frac{1}{N_{\mathrm{sam}}}\sum_{k=1}^{N_{\mathrm{sam}}}\rho_{k}$

As shown in Figure 1b,c for a fixed integration time, the area enclosed by the pinched hysteresis loop is used as an indicator of the memristive response, since it is closely related to the memory effect [25]. As the photon loss probability increases, the enclosed loop area gradually decreases, indicating a progressive weakening of the nonlinear response of the PQM.

To further characterize this evolution and evaluate the potential memory content of the hysteresis loops, we employ the form factor [26]:

(6) $$F=4\pi \frac{A}{P^{2}},$$

where *A* and *P* denote the enclosed area and the perimeter of the hysteresis loop, respectively. Based on the hysteresis loops shown in Figure 1, Figure 1e summarizes the evolution of the form factor under different integration times and photon loss probabilities. As the photon loss probability increases, the form factor decreases monotonically, indicating a gradual reduction in the potential memory content of the hysteresis loops. This trend is particularly pronounced at shorter integration times. In particular, the form factor drops from 0.5912 to 0.3435 for $T_{\mathrm{int}}=0.5$ $T_{\mathrm{osc}}$, and from 0.5666 to 0.2071 for $T_{\mathrm{int}}=0.01$ $T_{\mathrm{osc}}$. These results indicate that photon loss significantly modifies the dynamical response of the PQM, with non-Markovian memory effects being more strongly suppressed at shorter integration times.

In addition to photon loss, we also consider the effect of phase fluctuations on the device dynamics. In the PQM considered here, the reflectivity *R* is explicitly determined by the phase θ in a way that $R\left(\theta \right)=\cos^{2}\left(\theta /2\right)$ [17]. Therefore, phase fluctuations introduced during the PQM evolution can modify reflectivity, the input–output relation, and the final output probability distribution. Here we focus on such phase fluctuations during the PQM evolution.

Following the phase-noise modeling adopted in previous work [27], we describe the phase fluctuations by Gaussian noise. By combining Equation (1) with the phase-dependent reflectivity relation $R\left(\theta \right)=\cos^{2}\left(\theta /2\right)$, the input–output relation in the presence of phase fluctuations can be expressed as

(7) $$\langle {\hat{n}}_{\mathrm{out},C}\left(t\right)\rangle =\left[1-\cos^{2}\frac{\theta (t)+\delta \theta (t)}{2}\right]\langle {\hat{n}}_{\mathrm{in},A}\left(t\right)\rangle .$$

In this model the instantaneous value of $\langle {\hat{n}}_{\mathrm{out},C}\left(t\right)\rangle$ is given by a particular phase fluctuation δθ(t). Next we assume that the distribution of phase fluctuations reads:

(8) $$p\left(\delta \theta \left(t\right)\right)=\frac{1}{\sigma \sqrt{2\pi }}e^{-\delta \theta {\left(t\right)}^{2}/2\sigma^{2}},$$

where σ is the width of the Gaussian distribution. To investigate the effect of phase fluctuations on the dynamics of a single PQM device, we employ a sampling-based noisy-evolution approach, as summarized in Algorithm 2. In each realization, a phase fluctuation $\delta \theta_{k}$ is sampled and incorporated into the PQM evolution gate $U_{\mathrm{PQM}}$, yielding the output state $|\psi_{k}\rangle$. The corresponding density matrix is then constructed as $\rho_{k}\;=\;|\psi_{k}\rangle \langle \psi_{k}|$. By averaging over $N_{\mathrm{sam}}$ realizations, we obtain the final output density matrix $\rho^{\prime }$.
**Algorithm 2** Single-PQM Simulation Under Phase fluctuations.**Require:** Initial state $|\psi_{0}\rangle$, PQM evolution gate $U_{\mathrm{PQM}}$, number of samples $N_{\mathrm{sam}}$**Ensure:** Finally output density matrix $\rho^{\prime }$   1: **for**
k=1 to $N_{\mathrm{sam}}$ **do**   2:    sample a phase fluctuation $\delta \theta_{k}$ from the Gaussian distribution   3:    compute $|\psi_{k}\rangle \;=\;U_{\mathrm{PQM}}\left(\theta +\delta \theta_{k}\right)|\psi_{0}\rangle$   4:    compute $\rho_{k}\;=\;|\psi_{k}\rangle \langle \psi_{k}|$   5: **end for**   6: $\rho^{\prime }=\frac{1}{N_{\mathrm{sam}}}\sum_{k=1}^{N_{\mathrm{sam}}}\rho_{k}$

**Figure 3:**
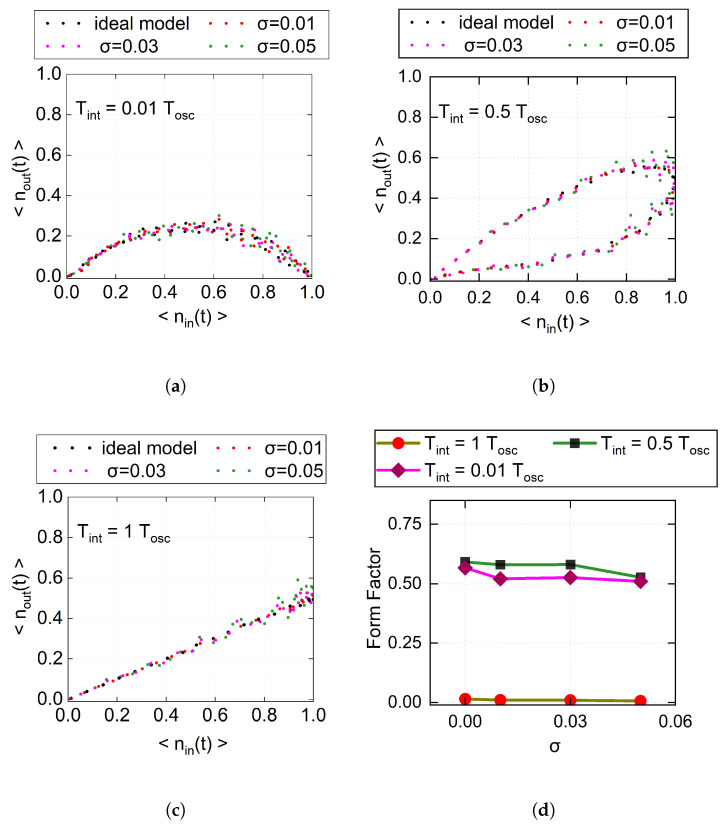
Effect of phase fluctuations on the dynamics of a single PQM. (**a**–**c**) Response curves under different integration times, where each panel compares the ideal model with the cases under different phase noise levels characterized by the standard deviation σ=0.01, 0.03, and 0.05. (**d**) Form factor as a function of σ for different integration times, where the brown, green, and purple curves denote $T_{\mathrm{int}}=T_{\mathrm{osc}}$, $T_{\mathrm{int}}=0.5\;T_{\mathrm{osc}}$, and $T_{\mathrm{int}}=0.01\;T_{\mathrm{osc}}$, respectively.

To investigate the effect of phase fluctuations on the device dynamics, we consider several representative values of the phase-noise standard deviation in the simulations, namely σ= 0.01, 0.03, and 0.05 [28]. Figure 3 shows the hysteresis loops under different integration times and phase noise levels. As σ increases, the loop shape changes only slightly, indicating that the device dynamics remain relatively robust against phase fluctuations within the considered noise range. To quantify this behavior, we further analyze the form factor defined in Equation (6), as summarized in Figure 3d. For all integration times considered, the form factor exhibits only a weak dependence on σ. In particular, for $T_{\mathrm{int}}=0.5$ $T_{\mathrm{osc}}$, the form factor shows only a slight decrease, from about 0.5912 to 0.5068, as σ increases from 0 to 0.05. Similarly, for $T_{\mathrm{int}}=0.01$ $T_{\mathrm{osc}}$, it decreases from about 0.5666 to 0.5097. By contrast, for $T_{\mathrm{int}}=1$ $T_{\mathrm{osc}}$, the form factor remains close to zero throughout the entire range of σ. These results indicate that phase fluctuations only mildly modify the dynamical response of the PQM and lead to a limited reduction in the potential memory content of the hysteresis loops.

**NARMA2 task.** Based on the previous analysis of a single PQM under photon loss and phase perturbation conditions, we find that photon loss has a more pronounced impact on the device dynamics. To further investigate the impact of device-level degradation on system-level information-processing performance, we construct a network composed of two PQMs. Previous work has shown that even small PQM networks can achieve strong performance on time-series prediction tasks [17]. We therefore use the NARMA2 task [29], a standard benchmark for nonlinear time-series prediction, to evaluate the effect of photon loss on the nonlinear processing capability of the PQM network. In our framework, the PQM network is designed to provide the nonlinearity and memory required for temporal information processing, while the linear readout layer is introduced only as a minimal post-processing module to map the quantum features to the target output. This design allows us to separate the role of quantum dynamical processing from that of final output mapping more clearly. Therefore, the predictive capability in the NARMA2 task mainly reflects the information-processing capacity of the PQM network, while the classical readout primarily serves as an output-mapping layer. The NARMA2 task considered here, y(k+1) depends on the current input u(k), the current output y(k), and the previous output y(k−1), and is given by

(9) $$y\left(k+1\right)=0.4y\left(k\right)\left(1+y\left(k-1\right)\right)+0.6u{\left(k\right)}^{3}+0.1,$$

where u(k) is a random input uniformly distributed over the interval [0,0.5].

To enable the quantum system to perform this task, an effective classical-to-quantum data encoding mechanism is required. In this work, amplitude encoding is adopted to encode the classical information x(n). As shown in the schematic of a single PQM in Figure 1a, port A serves as the input port. Accordingly, in the two-PQM network considered here, the classical input x(n) is encoded into a two-qubit separable state, and the two qubits are then injected into input ports A and $A^{\prime }$ of the two PQMs, respectively:

(10) $$|\psi_{\mathrm{in}}\rangle \;=\;{(\sqrt{x(n)}|0\rangle }_{A}+\sqrt{1-x(n)}{|1\rangle }_{A})⊗{(\sqrt{x(n)}|0\rangle }_{A^{\prime }}+\sqrt{1-x(n)}{|1\rangle }_{A^{\prime }}).$$

After the encoded information is fed into the quantum memristor network, quantum state tomography is performed at the output end, and the diagonal elements of the density matrix are extracted as the feature vector. The final prediction task is completed through a linear readout layer. In the linear readout layer, let there exist a weight matrix $W_{\mathrm{out}}$ for mapping the feature vector to the target output. The objective of the training process is to determine the optimal $W_{\mathrm{out}}$ by minimizing the error between the output vector and the true target vector. In this work, this procedure is implemented using linear regression. Specifically, the characteristic vectors obtained by inputting each training sample x(n) at each time step are assembled into a response matrix *X*, while the corresponding target outputs are arranged into a target matrix $Y_{\mathrm{target}}$. Then, the weight matrix $W_{\mathrm{out}}$ can be expressed as

(11) $$Y_{\mathrm{target}}=W_{\mathrm{out}}.$$

The model performance is evaluated using NMSE [30] as the primary metric, which is defined as

(12) $$\mathrm{NMSE}=\frac{\sum_{i=1}^{N}{\left(Y_{i,\mathrm{test}}-{\hat{Y}}_{i,\mathrm{test}}\right)}^{2}}{\sum_{i=1}^{N}Y_{i,\mathrm{test}}^{\;2}},$$

where $Y_{i,\mathrm{test}}$ denotes the true output value of the *i*-th sample in the test set, and ${\hat{Y}}_{i,\mathrm{test}}$ denotes the corresponding predicted value. This metric effectively characterizes the accuracy and robustness of the PQM model in the NARMA2 time-series prediction task and thus provides a quantitative benchmark for system optimization.

**Figure 4:**
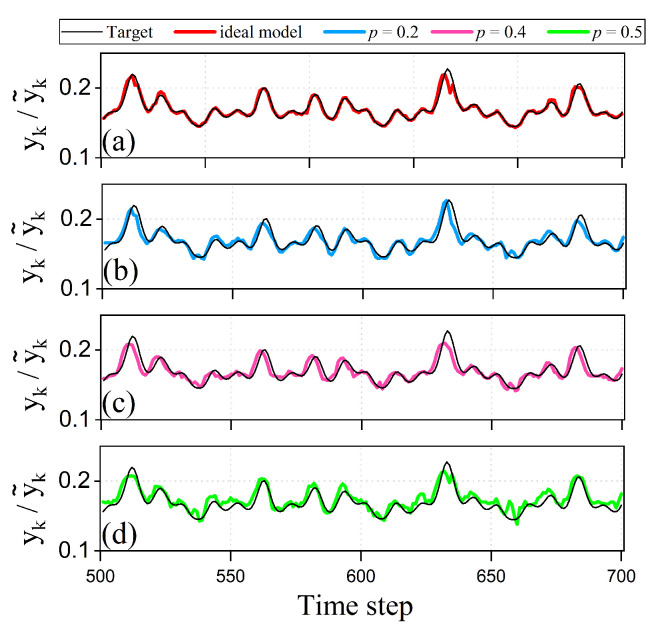
Prediction results of the quantum memristive network on the NARMA2 task under a fixed memory length setting, where the memory lengths of both memristors are set $T_{\mathrm{int}}$ to 2. (**a**) corresponds to the ideal model. (**b**–**d**) correspond to the models with different photon loss probabilities, namely p=0.2,p=0.4, and p=0.5. The dataset used contains 1000 data points, of which the first 500 are used for training and the remaining points for validation. For sake of clarity, only 200 data points from the test set are shown in the figure.

Figure 4 presents the prediction results of the PQM network on the NARMA2 task under both noiseless and photon loss conditions. The results show that the system performance is highly sensitive to photon loss, with a clear discrepancy between the prediction performance under noiseless and photon loss conditions. Under noiseless conditions, the PQM network can effectively preserve the nonlinear dynamic response and memory characteristics of the device, thereby exhibiting strong representational capability for the input samples, with an NMSE as low as 0.0448. In contrast, when photon loss is introduced, the prediction performance of the system deteriorates significantly. When the photon loss probability *p* is set to 0.2, 0.4, and 0.5, the NMSE increases to 0.1552, 0.2567, and 0.3056, respectively. The results show that higher photon loss probabilities lead to large prediction errors, indicating that photon loss weakens the network’s ability to represent and process input information and thereby reduces its task execution accuracy. Combined with the previous analysis of the single-device dynamics, these findings indicate that photon loss not only impairs the dynamical response of individual PQMs but also weakens the representational capacity at the network level, ultimately reducing task execution accuracy.

**Figure 5:**
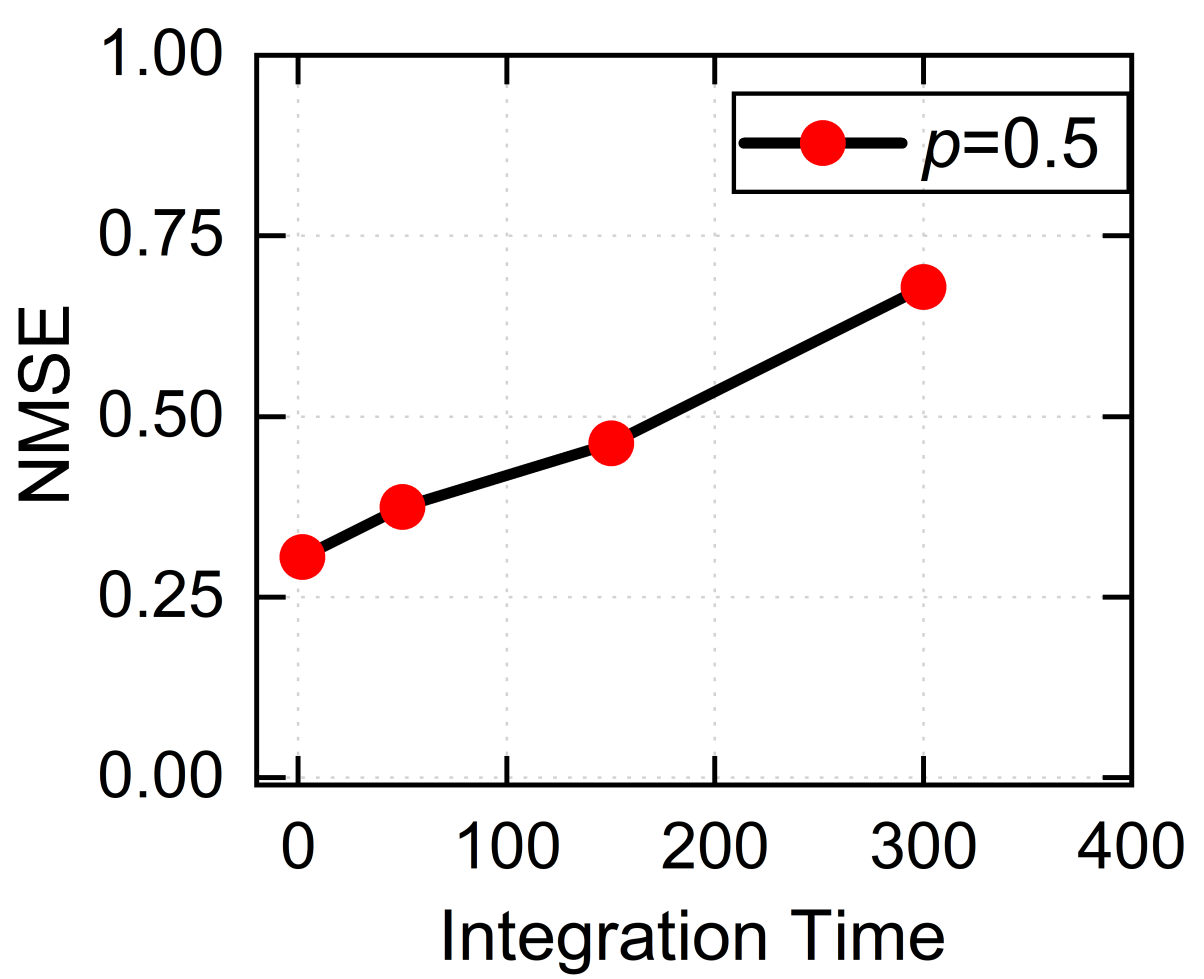
NMSE of the NARMA2 task versus integration time at a high photon loss probability *p*=0.5. The monotonic increase of the NMSE indicates that increasing the integration time under strong photon loss worsens the prediction performance.

To further investigate the effect of integration time on task performance under strong photon loss, we examine the NARMA2 prediction error at a high photon loss probability, p=0.5, for different integration times. In the NARMA2 task, $T_{\mathrm{int}}$ is chosen relative to the temporal scale of the input signal sequence. As shown in Figure 5, the NMSE shows an increasing trend as the integration time increases. Specifically, the NMSE rises from 0.3056 at $T_{\mathrm{int}}=2$ to 0.6792 at $T_{\mathrm{int}}=300$. Since the NARMA2 task is commonly used to evaluate the nonlinear processing capability of a system, this result indicates that increasing the integration time under strong photon loss weakens the nonlinear processing capability of the PQM network and consequently degrades prediction performance.

## 3. Conclusions

In this work, we investigate the hardware noise modeling and task performance evaluation of PQMs and their networked systems under incoherent evolution, predominantly induced by photon losses and phase fluctuations. First, for a single PQM, we employ the noisy gates approach to integrate dissipative effects directly into the device dynamics, comparatively analyzing the history-dependent input–output relations under varying photon loss probabilities, phase noise levels, and integration times. The results demonstrate that photon loss severely alters the dynamical evolution of the device, leading to evident deformations in its signature pinched hysteresis loops and degrading its non-Markovian memory properties.

Building upon this foundation, we construct a network composed of two independent PQMs and employ it in the NARMA2 time-series prediction task to evaluate the impact of such noise sources on the system’s information-processing capability. Under noiseless conditions, the memristive network effectively preserves the history-dependent dynamics, exhibiting strong representational capability for input samples with a NMSE of 0.0448. In contrast, the introduction of photon loss significantly degrades the prediction performance; for photon loss probabilities of 0.2, 0.4, and 0.5, the NMSE increases to 0.1552, 0.2567, and 0.3056, respectively. Moreover, at a high photon loss probability of 0.5, the NMSE further increases from 0.3056 at $T_{\mathrm{int}}=2$ to 0.6792 at $T_{\mathrm{int}}=300$, indicating that extending the integration time fails to compensate for the accuracy loss caused by high photon dissipation. These findings indicate that photon loss not only impairs the non-Markovian dynamics of individual PQMs but also weakens the representational capacity at the network level, ultimately reducing task execution accuracy.

Consequently, beyond optimizing the physical hardware of individual devices, future practical deployments must prioritize the design of loss-tolerant network architectures and system-level robustness. In this architecture, photon dissipation is localized to individual device evolutions rather than accumulated across the entire network [31]. Specifically, the building blocks of PQM involve only single-qubit operations, classically controlled single-qubit operations, and measurements. This provides a promising direction for future work on loss-tolerant PQM architectures. Future work may incorporate additional noise sources, such as imperfect single-photon indistinguishability, to investigate their coupled effects on the task performance of PQM networks, thereby offering comprehensive guidance for scalable system optimization.

## Data Availability

The code used in this paper is available upon request.
